# Breast diseases in women over the age of 65 in Monastir, Tunisia

**DOI:** 10.11604/pamj.2018.31.67.16105

**Published:** 2018-10-01

**Authors:** Anis Haddad, Olfa Zoukar, Amira Daldoul, Hanene Bhiri, Khechine Wiem, Houda Mhabrich, Sonia Zaied, Raja Faleh

**Affiliations:** 1 Department of Gynecology and Obstetrics, El Omrane Hospital, Monastir, Tunisia; 2 Department of Oncology, Fattouma Bourguiba University Hospital, Monastir, Tunisia; 3 Department of Oncology, Farhat Hached University Hospital of Sousse, Tunisia; 4 Department of Radiology, El Omrane Hospital, Monastir, Tunisia

**Keywords:** Age, breast neoplasms, breast diseases, surgical procedures

## Abstract

As life expectancy is on the rise, it is predicted that a growing number of people will live beyond the age of 65 and therefore a higher number of elderly women will have breast diseases requiring significant health care and services. This study is aimed at investigating the characteristics, the treatment and outcomes of women older than 65 years old treated for breast diseases at our institution. This was a retrospective study covering the period from January 2003 to December 2011. It involved 92 patients aged over 65 and treated for breast disease in the Maternity Center of Monastir, Tunisia. The data included characteristics of patients and tumors, treatment and outcomes that were obtained through data extraction sheets. We reported a study of 92 women over the age of 65 of whom 77 women had malignant breast disease (83.6%) and 15 benign breast diseases (16.4%). Breast cancer was discovered at a mean age of 72.5 ± 6.6 years. Distant metastases were found in 5.3% of cases and infiltrative ductal carcinoma was detected in 85.7% of patients. Hormonal receptors were positive for estrogens in 64.7% of cases. Surgical treatment was performed in 73 patients and adjuvant treatment was prescribed for 67 women (86%). The complication rate was 16.6% among the 73 patients who underwent surgery. Benign breast diseases represented 16.3% of the mammary pathologies. Abscesses and fibrocystic mastopathy were the most frequent histological diagnoses. Despite great interest in geriatric gynecological pathology worldwide, many questions related to how optimally treat this patient population remain unanswered. In this study, a surgical treatment was performed in 94.8% of breast cancer patients and the complication rate was 16.6%.

## Introduction

Nowadays, the health care of elderly people is of great interest. Population trends will result in a growing number of people over the age of 65. In addition, this age group is increasingly demanding and requires significant health care and services. In Tunisia, the prevalence of elderly people, which was 9% in 2003, reached 9.5% in 2009 and will reach 17% in 2029 [1]. Greater interest in gynecological pathology has been centered on sexually active woman than on elderly woman. This is underlined by health programs aimed primarily at promoting the health of mothers and children. To our knowledge, only a few aspects of pathologies of the breast in Tunisian elderly women have been dealt with.

## Methods

This was a retrospective study from January 2003 to December 2011. It involved 92 patients treated for breast diseases in the Maternity Center of Monastir and fulfilling the following inclusion criteria: aged over 65 and having malignant or benign breast disease. The data were obtained through data extraction sheets. The variables studied were: age at diagnosis, family and personal history, age of puberty, age of menopause, parity, time and reason for consultation, TNM stage, histological type and treatment. We included 92 patients: 77 breast cancer cases and 15 benign conditions. The statistical analyses were performed using the SPSS 18.0 statistical software package. Data analysis was descriptive in nature. Conventional formulae were used to calculate the means and standard deviations.

## Results

In the current study, breast pathology involved 92 women, including 77 breast cancer cases (83.6%) and 15 benign conditions (16.4%).

### Malignant breast diseases

The average age of patients with breast cancer was 72.5 ± 6.6 years [65 - 89 years]. This cancer affected mainly the 65-74 age group. Four women (5.2%) had a family history of breast cancer. Of the 77 women in this group, 4 women had precocious puberty (< 12 years). The mean age at puberty was 13.1 ± 1.4 years [10 - 16 years]. Parity for the breast cancer group was very close to that of all the series. Indeed, the average parity was 5.8 ± 3.5 [0 - 17]. In addition, nulliparity was found in 12 women (15.6%). The absence of breastfeeding was noted for 14 women (18.2%) and was not specified for 9 women. The mean age at menopause was 49.4 ± 4.4 years [38 - 57]. Late menopause onset (> 55 years) was noted in 5 women (6.5%) and 2 women had used hormonal contraception. A total of 61 women (79.2%) with breast cancer had a history of high blood pressure and diabetes. In addition, in these women, a surgical history was reported in 31 women (40.2%). We particularly noted 3 cancers, 5 breast abscesses and a breast adenofibroma.

For breast cancer, the palpable mass was the chief complaint. It was found in 68 women followed by mastitis and nipple discharge in 8 and 5 women respectively. The average consultation delay was 5.2 ± 5 months [0 - 16 months]. This time was calculated for 76 women. The most represented T stage for breast cancer was T2 according to the TNM classification followed by T4 with respective rates of 54% and 38%. It should be noted that one woman had bilateral involvement: T2 on one side and T1 on the other (Table 1). For breast cancer, N1 stage of nodal invasion was reported in 40 women (51.9%) followed by N0 and N2 in 35(45.4%) and 2 (2.7%) women respectively. The woman with bilateral involvement had N0 on one side and N1 on the other (Table 1).

**Table 1:** Details of the TNM staging system

T categories for breast cancer	
TX:	Primary tumor cannot be assessed.
T0:	No evidence of primary tumor.
T1:	Tumor is 2 cm or less across.
T2:	Tumor is more than 2 cm but not more than 5 cm across.
T3:	Tumor is more than 5 cm across.
T4:	Tumor of any size growing into the chest wall or skin. This includes inflammatory breast cancer.
**N categories for breast cancer**	
NX:	Nearby lymph nodes cannot be assessed (for example, if they were removed previously).
N0:	Cancer has not spread to nearby lymph nodes.
N1:	Metastases to movable ipsilateral axillary lymph node(s)
N2:	Metastases in ipsilateral axillary lymph nodes that are clinically fixed or matted; or in clinically detected ipsilateral internal mammary nodes in the absence of clinically evident axillary lymph node metastases
N3:	Metastases in ipsilateral infraclavicular lymph node(s) with or without axillary lymph node involvement; or in clinically detected ipsilateral internal mammary lymph node(s) with clinically evident axillary lymph node metastases; or metastases in ipsilateral supraclavicular lymph node(s) with or without axillary or internal mammary lymph node involvement
**M categories for breast cancer**	
M0:	No clinical or radiographic evidence of distant metastases
M1:	Distant detectable metastases as determined by classic clinical and radiographic means and/or histologically proven larger than 0.2 mm

At the time of diagnosis, 4 patients (5.3%) had distant metastases. The most common histological type was invasive ductal carcinoma (IDC) detected in 68 women (87.2%). The histoprognostic grade was specified in 75 women. It was grade I for 38.7% of women and II for 38.7%. The hormone receptor study was performed in 74 cases: it was positive estrogen receptors (ER+) in 47 cases (63.5%) and positive progesterone receptors (PR+) in 36 cases (48.6%). A surgical treatment was performed on 73 patients: surgery was first in 65 women (83.3%) and it followed neo-adjuvant chemotherapy for 8 patients whose tumor was diagnosed at an advanced stage.

Patey’s mastectomy with nodal dissection was performed in 54 cases (70.1%). A conservative treatment was performed in 6 patients including a woman with a bilateral cancer treated on the left with Patey’s mastectomy and on the right by a simple lumpectomy. A palliative mastectomy was performed in 13 cases (16.6%). Adjuvant therapy was prescribed for 67 women (87%). This included radiotherapy for 41 (61.2%) women, chemotherapy for 48 (71.6%) women and hormone therapy for 24 (35.8%) women according to different protocols. Among the 73 patients who underwent surgery, the follow-up was simple for 60 cases. The sequels were mainly dominated by infectious complications. One death was reported in a 76 year old female patient (hypertensive and hemiplegic) on day 4 after cardiac failure. After an average follow-up of 4.5 years, 5 patients had locoregional recurrence with or without axillary involvement. A case of distant metastasis was also observed.

### Benign breast diseases

As for benign breast diseases, they represented 16.3% of the mammary pathologies. Fibrocystic mastopathy and abscesses were the most frequent histological diagnoses. The mean age of patients in this group was 68.2 ± 3.1 years [65 -75 years]. The main surgical procedures varied according to the indications. These consisted of three tumorectomies, three pyramidectomies, two zonectomies and three biopsies. A flattening under antibiotic cover was performed for the 4 breast abscesses of our series. The operative follow-ups were simple in the majority of cases. A single hematoma case of the scar was observed.

## Discussion

Due to the epidemiological characteristics including population aging, the rise in life expectancy as well as the increasing incidence of cancer with age, elderly to very old women represent a large proportion of breast cancer patients. The U.N. population division estimates one in five persons are expected to be 65 or older by 2035 in the United States. In addition, data from the Surveillance Epidemiology and End Results Program indicate that the incidence of breast cancer increases up to 80 years of age and plateaus between 80 and 85 years of age [2]. For elderly women in Europe, the incidence of breast cancer is 320/100,000 women between 65 and 75 years old and 300/100,000 women after the age of 80 [3]. Like all cancers, the frequency of breast cancer increases with age [4]. The authors reported that 45% of breast cancers were diagnosed in women over 65 [5]. The nodule or palpable mass is the most frequent reason for consultation. In the Chatzidaki *et al.* series [6], palpable nodule and nipple discharge were the reasons for consultation in 58.5% and 7.5% of cases. In the Jedidi series [7], the most common reasons for consultation were palpable nodule (77.9%), skin changes (22%) and nipple discharge (10.3%). In our series, the most common reasons for consultation were palpable nodule (89.5%), carcinomatous mastitis (10.5%) and nipple discharge (6.6%). There is a consultation delay of more than 3 months or more for elderly women with breast cancer [8]. In this age group, this delay is mainly due to the absence of similar cases in the family, the absence of a partner or the fear of the consequences of cancer [8]. In the Jedidi series [7], the average consultation delay was 11.2 months in Sfax Hospital in the south of Tunisia.

In our series, the average consultation delay was shorter (5.2 months) and this can be explained by the fact that Sfax Hospitals admitted more women from the inner regions. In fact, in the inner regions, there are poor information campaigns and low awareness of health issues among women. To reduce this delay and improve the prognosis of breast cancer, Forbes *et al.* [9] offer elderly women an education program that involves the following objectives: To recognize the symptoms of breast cancer, identify the risk factors for breast cancer, encourage early consultation, discuss the benefits of early consultation and reduce the fear of the consequences of breast cancer. The size of the tumor is a prognostic factor. The literature reports that 45-80% of elderly patients present with T2 or greater or palpable tumors [10]. In our series and those of Jedidi [7], tumors classified T2 are more frequent followed by those classified T4 (Table 1). One would hypothesize that the lack of standard screening guidelines for elderly women may lead to advanced disease at diagnosis. Some studies have reported that elderly women over the age of 80 have a higher likelihood of presenting with advanced disease [11], with up to 44% axillary nodal involvement [12], or with breast cancers that have poor prognostic features [12]. However, other studies suggest that older women have less aggressive disease [13, 14] and are less likely to have nodal involvement [15].

Like those of Jedidi [7], our series revealed that node invasion was mainly of classes N0 and N1 (Table 1). Infiltrating ductal carcinoma remains the most common histological subtype of breast cancer diagnosed in older and younger patients. Older patients are reported to have a greater frequency of tumors with more indolent histologies and an overall more favorable biological tumor profile [16]. This profile is characterized by a higher percentage of estrogen receptor-positive (ER) tumors (83% ER in patients under 65, 87% to 91% ER tumors in patients 65 and older) [12]. The proportion of ER tumors continues to rise even within the over-65 cohort, with 87% of patients aged 65 to 74 years having ER tumors, compared with 91% of patients aged 85 and older. Reduced proliferation markers (such as S-phase fraction) and HER2/neu negativity are also features of breast tumors in the elderly [12]. In our series we found RE + in 64.7% of cases and RP+ in 48.6% of cases. The gerontological community has long recognized the heterogeneity within the elder patient group. The concept of chronological versus physiological age is difficult to quantify, yet is of inherent importance in clinical decision-making. Aging affects multiple body systems and remains an individualized process that correlates poorly with chronological age. The concept of “functional age” has thus emerged. Two surrogate markers of functional age, comorbidities and the gerontological assessment, are currently used in cancer care-related decisions. The present recommendations by the National Comprehensive Cancer Network (NCCN) include the use of a geriatric assessment tool in developing care plans for all cancer patients aged 70 and older [17]. No current assessment tool has emerged as the preferred choice.

Today, the majority of breast cancer treatment recommendations appear to be modeled on younger patients, as most clinical trials focus on healthy, young women [18]. Several authors agree that older women should have the same treatment options as young women [19]. Survival without recurrence and/or without metastasis and the relative risk of mortality depend on a good indication for adjuvant therapy, the presence of prognostic factors represented mainly by SBR grade, lymph node involvement, tumor size and the presence of hormonal receptors. The management of the mammary pathology in the elderly patient is often suboptimal with a general tendency to under-treatment. It is characterized not only by a decrease of the adjuvant therapeutic indications but also by an increase in abstentions and a reduction in palliative care, analgesic and comfort management [20]. In the elderly, it is difficult to evaluate the indication of adjuvant chemotherapy whose toxicities seem to be accentuated with age.

Surgery is still the cornerstone in the treatment of early-stage breast cancer. Several studies have shown that breast conservation surgery followed by radiation therapy is as effective as more extensive surgery options such as mastectomy [21]. Nevertheless, some studies suggest that elderly women are less likely to be offered breast conservation surgery than their younger counterparts [22, 23]. Partial surgery would be better tolerated in the short and medium terms on the aesthetic, functional and psycho-cognitive aspects [24]. For those who have a lot of co-morbidities, surgery under local anesthesia may be better tolerated than general anesthesia especially in cases of severe psycho-cognitive disorders [19]. Perioperative morbidity is low and mortality ranges from 0 to 2%, which is due to co-morbidities and not to chronological age [19]. In patients with hormone receptor-negative breast cancer, chemotherapy has been proven to have a survival benefit. A growing body of data suggests that chemotherapy also leads to improved survival in elderly patients [25]. Clinical trials show that radiotherapy after breast conserving surgery reduces breast cancer recurrence among older women with early-stage disease [26]. However, survival as well as local control is likely to be diminished for older women with life expectancies of 10 years or more with large or node-positive tumors who do not receive radiation therapy [27].

In contrast to the trends in breast surgical procedures, elderly women are less likely to undergo axillary staging procedures compared to younger women [12, 13]. This is despite previous studies demonstrating that a nodal involvement retains prognostic significance in older patients with breast cancer [28] and that adjuvant treatment decisions are altered in many elderly patients based on axillary staging results [29]. Whether the absence of axillary staging necessarily results in less favorable outcomes is not very clear. Martelli *et al.* [30] reported a 5-year follow-up on a cohort of 219 women aged 65-80 with clinical T1N0 breast cancer who were randomized to axillary lymph node dissection versus no axillary surgery. Two women who had no axillary surgery developed an axillary recurrence, but there was no difference in overall or disease-specific mortality between the two groups [30]. Mandelblatt *et al.* [31] found that women aged 67 and older who underwent axillary staging, either by sentinel lymph node dissection or axillary lymph node dissection, developed three times more upper extremity complications than younger women. Those sequelae also had a larger impact on physical and mental function in the older patients [31]. The risks of axillary procedures in elderly women must be weighed against the potential prognostic information and benefits obtained. This is true for women of all ages, but may be particularly relevant in the elderly population. In the conclusions of the AMAROS [32] study, the authors considered that radiotherapy in elderly women with sentinel nodules is a good alternative in order to avoid morbidity associated with ganglion dissection.

## Conclusion

In this study, the average age of breast cancer patients was 72.5 years, the palpable mass was the main complaint and the most common histological type was invasive ductal carcinoma. Even if the interest in geriatric gynecological pathology started early in Western countries, many questions remain unanswered on how to optimally treat this patient population. In this study, a surgical treatment was performed in 94.8% of breast cancer patients and adjuvant treatment was prescribed for 87% of women, although there is a lack of good quality evidence regarding the role of adjuvant chemotherapy.

### What is known about this topic

- 45% of breast cancers are diagnosed in women over 65;
- Only few aspects of breast pathologies of the elderly women in Tunisia have been treated.

### What this study adds

- Breast cancer affected mainly the 65-74 years old age group;
- The palpable mass was the main chief complaint;
- Even if data suggest that older women could not have aggressive treatment and that standard treatments cause more complications in elderly patients, surgical treatment was performed in 94.8% of breast cancer patients.

## Competing interests

The authors declare no competing interests.
